# Retraction: LncRNA TCF7 promotes epithelial ovarian cancer viability, mobility and stemness via regulating ITGB8

**DOI:** 10.3389/fonc.2023.1307324

**Published:** 2023-10-19

**Authors:** Frontiers Editorial Office

The Publisher retracts the cited article.

Following publication, the publisher has discovered that the authors provided false information for the peer-review process. As the scientific integrity of the article cannot be guaranteed, and adhering to the recommendations of the Committee on Publication Ethics (COPE), the publisher therefore retracts the article.

The authors have not responded to this retraction.

This retraction was approved by the Chief Editors of Frontiers in Oncology and the Chief Executive Editor of Frontiers.

